# A Novel Mobile App-based Neuromuscular Electrical Stimulation Therapy for the Management of Knee Osteoarthritis: Results From an Extension Study of a Randomized, Double-blind, Sham-controlled, Multicenter Trial

**DOI:** 10.5435/JAAOSGlobal-D-22-00115

**Published:** 2022-09-12

**Authors:** Vinod Dasa, Nebojsa V. Skrepnik, Dena Petersen, Ronald E. Delanois

**Affiliations:** From the Department of Orthopaedic Surgery, LSU Health Sciences Center, New Orleans, LA (Dr. Dasa); the Tucson Orthopaedic Institute, Tucson, AZ (Dr. Skrepnik); the Noble Clinical Research, Tucson, AZ (Dr. Petersen); and the Rubin Institute for Advanced Orthopedics, Center for Joint Preservation and Replacement, Sinai Hospital of Baltimore, Baltimore, MD (Dr. Delanois).

## Abstract

Mobile app-based neuromuscular electrical stimulation (NMES) is a promising treatment of knee osteoarthritis as previously demonstrated in a 12-week, randomized, double-blind, sham-controlled, multicenter trial (parent study).

**Methods:**

Sixty-four of the 253 patients with knee osteoarthrosis who completed the 12-week parent study were enrolled in a 14-week extension study during which they continued to receive double-blind, home-based NMES (two 20-minute daily sessions, 5 d/wk) with either the original device (“active NMES”) or a low-voltage version (“sham NMES”). All subjects who enrolled in the extension study comprised the intent-to-treat population and subjects who applied NMES (compliance monitored through the mobile app and a remote portal) for at least 2,800 minutes (14-week device usage) comprised the per-protocol therapy compliant population.

**Results:**

In the per-protocol therapy compliant population, the active NMES group (n = 21) had a higher reduction in Visual Analog Scale Nominated Activity (64.7% versus 24.3%, *P* = 0.020) and Visual Analog Scale Nominated Activity improvement ≥50% (76.2% versus 12.5%, *P* = 0.002) than the sham NMES group (n = 8). Outcomes were not markedly different between groups in the intent-to-treat population.

**Discussion:**

Applying NMES therapy for an additional 14 weeks (totaling 26 weeks) resulted in notable and clinically meaningful pain relief when patients were fully compliant with NMES.

Knee osteoarthritis (OA) is a leading cause of disability and poses a substantial health and economic burden in the United States.^1^ In 2007 to 2008, 14 million adults in the United States had symptomatic knee OA and this number was projected to increase due to an aging population and the growing prevalence of obesity, a primary risk factor for knee OA.^2^ The etiology of knee OA is not well understood but believed to result from the complex interplay of constitutional and mechanical factors, including joint integrity, genetic predisposition, local inflammation, mechanical forces, and cellular and biochemical processes.^3^

Quadriceps muscle weakness contributes to the onset and progression of knee OA^4,5,6,7,8,9,10^ by decreasing proprioception, joint stabilization, and shock absorption.^9,11,12^ Quadriceps weakness emerges before patient-reported symptoms in patients with radiographic knee OA^9^ and is associated with increased pain and disability in patients with knee OA.^13,14^ Exercise is a universally recommended component of knee OA management,^15,16^ and exercise designed to strengthen the quadriceps muscle reduces OA-related knee pain and disability.^17,18^ Unfortunately, knee pain and stiffness as well as psychological factors prevent many patients with knee OA from engaging in exercise/activity,^19,20^ which may contribute to a vicious cycle of additional muscle weakness, joint deterioration, and more severe pain and disability.^5^ Furthermore, in severe knee OA, deficits in volitional muscle activation contribute more to quadriceps weakness than muscle atrophy, which may limit the effectiveness of therapies relying on volitional muscle activation.^21^

Neuromuscular electrical stimulation (NMES) is an emerging method of quadriceps muscle strengthening that does not rely on patient exercise or voluntary muscle contractions. NMES sends low-level electrical impulses that cause involuntary contractions in targeted muscles and preferentially recruits and activates type II muscle fibers in the target muscle group.^22^ Randomized clinical trials have shown that NMES plus physical therapy increases quadriceps strength, reduces pain, and improves physical function during the first 4 to 12 weeks after total knee arthroplasty and anterior cruciate ligament reconstruction compared with physical therapy alone.^23,24,25,26,27^

Comparably fewer studies of NMES have been conducted in the setting of knee OA. Reviews of studies of NMES for knee OA have concluded that results generally support the effectiveness of this modality for improving muscle strength and reducing pain, but that research has been limited by the use of concurrent treatments (eg, physical therapy/exercise) that may improve quadriceps strength, methodological limitations, and widely variable NMES parameters and treatment duration.^28-30^

Home-based NMES, a more convenient and lower cost means of delivering therapy than clinic-based NMES, has been shown to be feasible and effective in the settings of preoperative and postoperative total knee arthroplasty^25,26,31,32^ and knee OA.^33,34^ A recent randomized, double-blind, sham-controlled, multicenter study demonstrated that CyMedica Orthopedics home-based NMES therapy (CyMedica Orthopedics) produced clinically meaningful improvements in knee pain relief, joint stiffness, and joint function after 12 weeks.^35^ In this study, 156 patients with knee OA were randomized 2:1 to the original home-based NMES therapy or sham-modified low-voltage NMES therapy for 12 weeks. In the intent-to-treat (ITT) population, the primary outcome, percentage change from baseline in the Visual Analog Scale (VAS) pain for a patient-nominated physical activity (VAS Nominated Activity), was not statistically significant between groups, and the active NMES group achieved a significantly larger reduction in the Western Ontario and McMaster Universities Osteoarthritis Index (WOMAC) Pain (−36.8% versus −26.6%, *P* = 0.038), Stiffness (−44.7% versus −17.4%, *P* = 0.010), and Function (−40.1% versus −24.5%, *P* = 0.029) subscales. Quadriceps strength was significantly improved from baseline only in the active NMES therapy group (active NMES: 64.9%, *P* = 0.0001; sham NMES: 56.3%, *P* = 0.0643). Improvements in patient-reported outcomes and quadriceps strength were most pronounced when analyses were conducted in the per-protocol therapy compliant (per-protocol therapy compliant) population, which included only active NMES therapy patients who adhered to the full daily treatment dose of two sessions per day (20 minutes each), 5 days of the week. The purpose of this study was to report outcomes of pain, stiffness, and function in patients who completed the 12-week parent study described above and continued receiving double-blind treatment for an additional 14 weeks. We hypothesized that subjects who continued to receive active NMES therapy would achieve greater improvements in knee pain and symptoms than those who received sham NMES therapy, with the largest benefit obtained by subjects who were fully adherent to therapy.

## Methods

### Study Design and Patients

In the multicenter, double-blind, sham-controlled parent study (NCT04128618), patients with symptomatic (pain VAS ≥4), degenerative knee OA (Kellgren-Lawrence grade II, III, or IV) were randomized 2:1 to receive the original, active NMES therapy (“active NMES”) or a modified low-voltage version of NMES therapy (“sham NMES”) for 12 weeks. A complete list of inclusion and exclusion criteria is given in Supplemental Digital Content 1, http://links.lww.com/JG9/A226. Patients who completed the parent study were invited to enroll in a 14-week extension study during which they would continue to receive the same double-blind treatment as they received in the parent study. Because the extension study was not initiated until after 78 of the 159 patients had completed the parent study, these 78 patients were ineligible to enroll in the extension study. Central or local Institutional Review Board approvals were obtained for each site in accordance with the International Council for Harmonisation Guidelines for Good Clinical Practice. All patients provided written informed consent before participation.

### Study Interventions

**Figure 1:**
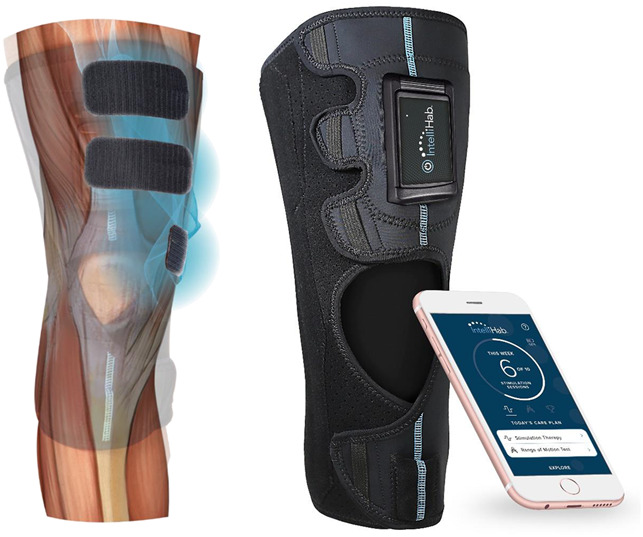
Photograph showing the home-based NMES system (see figure on right). The NMES treatment is applied using prepositioned electrodes inside the knee garment that would be placed on the vastus medialis oblique and rectus femoris muscles of quadriceps (see the figure on left). NMES = neuromuscular electrical stimulation

The CyMedica NMES system (CyMedica Orthopedics) consists of a knee conductive garment with an incorporated controller (waveform pulse generator), docking receptacle, two range of motion sensors, and three electrodes (Figure 1). The electrodes were designed to be placed on the vastus medialis oblique and rectus femoris muscles of the quadriceps. A mobile app was used by the patients to deliver the therapy and included digital health features that facilitate the reporting of actual applied NMES therapy to a remote structured query language and Health Insurance Portability and Accountability Act compliant database.

Patients in the active NMES group received therapy using the original, active CyMedica NMES system (CyMedica Orthopedics), and those in the sham NMES group received therapy using a modified low-voltage version of the CyMedica NMES system (CyMedica Orthopedics). All patients were instructed to set NMES intensities for the knee and thigh at the highest tolerable limit. These intensities were adjustable by the patient and could range from level 0 (no voltage) to 100 (maximum available voltage). In the active NMES group, the root mean square nominal voltages associated with levels 1 to 100 ranged from 0.5 to 9.2 V for an impedance of 500 Ω and 0.5 to 16.4 V for an impedance of 2,000 Ω. Patients in the sham NMES group were given a modified version of the NMES system with a maximum applied intensity of level 5 (the associated root mean square nominal voltage for level 5 is 1.86 V for an impedance of 500 Ω and 3.18 V for an impedance of 2,000 Ω).

Patients in both study arms were told that they may or may not feel a muscle tingling or a muscle contraction during therapy. Both groups were instructed to apply NMES therapy twice daily (20 minutes each) 5 days a week (10 sessions for a total of 200 minutes per week) for 14 weeks (a minimum of 2,800 minutes of therapy over the 14-week study).

### Randomization and Blinding

Patients continued in the same randomization group as the parent study. A random permuted block methodology stratified by site had been used in the parent study. Assignment was in a 2:1 ratio of active NMES to sham NMES. Patients and researchers collecting patient measurements were blinded to the device type/treatment group. The investigators were blinded to patient treatment allocation.

### Outcome Measures

Assessments were conducted at baseline (up to 21 days from parent study completion) and at 14 weeks (±7 days). The home-based NMES treatment continued or restarted immediately after the baseline visit. Patient-reported outcome measures included VAS, WOMAC,^36^ and a 7-point Patient Global Impression of Change (PGIC). A single question assessed patient's satisfaction with the treatment on a five-point scale from 0 (very dissatisfied) to 4 (very dissatisfied). Although the Knee Osteoarthritis and Outcomes Score Jr was also used to evaluate self-reported knee symptoms because this questionnaire is largely redundant with the WOMAC, results for this scale were not analyzed.

VAS was assessed for pain for a patient nominated activity (VAS Nominated Activity)^24^ and a general knee pain (VAS General). VAS Nominated Activity was defined as a physical activity (eg, climbing stairs) that caused the worst knee pain for the patient at the parent study baseline visit; this same activity was used during the extension study assessments of VAS Nominated Activity. The 24-item WOMAC consists of three subscales—Pain (five items), Stiffness (two items), and Function (17 items)—and a total score representing general knee health.

Isometric quadriceps strength was assessed using a handheld dynamometer (model 01 163; Lafayette Instrument Company); peak torque (lb.ft) exerted from the patient's lower leg when pushed against the dynamometer represented the patient's isometric quadriceps strength.

Objective functional assessments included the Timed Up and Go (TUG) test and repeated the chair rise test. The 3-minute walk test was also administered but not analyzed. The TUG test measured the time it took a subject to rise from a chair, walk 3 m (9 ft 10 inches), turn, walk back to the chair, and sit down. In the repeated chair risk test, the number of stands a subject can complete from a seated position, without the use of arms, over 30 seconds are counted. At the week 14 visit and throughout the course of study, patients were asked if they had experienced an adverse event.

### Use of Concomitant Medications and Therapies

Prescription pain medications, knee injections, physical therapy, and unloader bracing treatment in the target knee were prohibited during the study duration. Patients could take acetaminophen or equivalent (up to 3,000 mg/d) as needed. Patients were asked to log their medication usage and stop taking pain medication 24 hours before each visit.

### Study End points

Efficacy end points were evaluated as change from the baseline (of the parent study) to week 14 of the extension study (week 26 overall). The primary end point was change in VAS Nominated Activity from baseline to week 14. When interpreting the results of clinical trials of interventions for chronic pain, a ≥50% reduction on a 0 to 10 pain VAS is considered a substantial improvement^37^ and defined a VAS Nominated Activity responder. Secondary end points included the VAS General, WOMAC, PGIC, and patient satisfaction. Responders for secondary end points assessed at baseline and week 14 were defined as patients who achieved ≥50% improvement. For the PGIC, a response of “much” or “very much” improved at week 14 was considered a clinically important change.^37^

### Statistical Analysis

Statistical analysis was conducted using SAS version 9.4 (SAS Institute). The ITT population was defined as all rolled-over patients. The per-protocol therapy compliant population was defined as all rolled-over patients with at least one session of study therapy applied, no major protocol deviations, and who applied the therapy for at least 2,800 minutes during the study.

Chi-square tests compared 50% responder rates for primary and secondary end points. The change from baseline to week 14 for each continuous end point was tested for normality using the Shapiro-Wilk test. If change from baseline was normally distributed, an independent two-sample Student *t* test was used to compare mean values between the treatment groups; if normality testing failed, the Mann-Whitney *U* test was used.

## Results

### Patient Demographic and Baseline Characteristics

**Figure 2:**
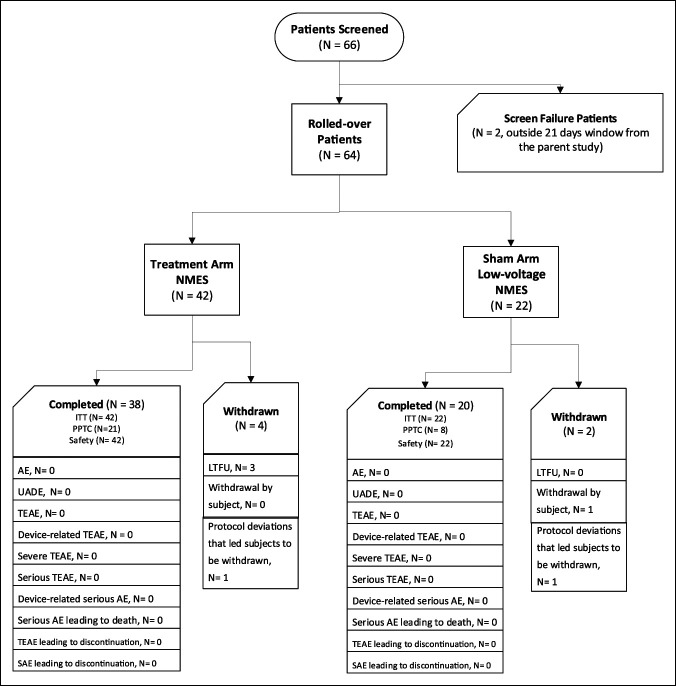
Flowchart showing the patient disposition. VAS = Visual Analog Scale

At the time of initiation of the extension study, 81 patients who had completed the parent study were eligible for enrollment, of whom 66 were screened and 64 (80% of the available subjects) were deemed eligible and enrolled in the extension study (Figure 2). The characteristics of the extension study sample were similar to those of the parent study sample, although no statistical comparisons were made. Table 1 summarizes the demographic and baseline characteristics in the ITT (n = 42) and per-protocol therapy compliant (n = 29) populations. Among all patients, 62% (42/64) were female and 74% (37/64) were White. The mean (SD) age and body mass index were 60.0 (10.4) years and 31.6 (6.0) kg/m^2^, respectively. The distribution of Kellgren-Lawrence grades was 33% grade 2, 20% grade 3, and 11% grade 4.

**Table 1:** Demographics and Baseline Characteristics of ITT and PPTC Populations

Characteristic	ITT Population		PPTC Population	
	Active NMES (n = 40)	Sham NMES (n = 22)	Active NMES (n = 21)	Sham NMES (n = 8)
Age, yr				
Mean (SD)	62.1 (9.93)	56.0 (10.29)	63.6 (9.23)	59 (11.7)
Median (range)	63.5 (34-79)	55.0 (41-72)	64.0 (42-79)	61.0 (41-72)
Sex, n (%)				
Female	16 (38.1)	6 (27.3)	8 (38.1)	3 (37.5)
Male	26 (61.9)	16 (72.7)	13 (61.9)	5 (62.5)
Race, n (%)				
White	26 (61.9)	15 (68.2)	17 (80.9)	5 (62.5)
Black	13 (31.0)	7 (31.8)	3 (14.3)	3 (37.5)
Asian	3 (7.1)	0	1 (4.8)	0
Others	1 (0.9)	0	0	0
Body mass index, kg/m^2^				
Mean (SD)	33.4 (6.4)	32.2 (6.4)	34.3 (5.8)	32.3 (7.1)
Median	33.0	33.5	34.5	31.4
Kellgren-Lawrence grade, n (%)				
2	20 (47.6)	13 (59.1)	8 (38.1)	5 (62.5)
3	12 (28.6)	8 (36.4)	6 (28.6)	3 (37.5)
4	10 (23.8)	1 (4.5)	7 (33.3)	0

ITT = intent-to-treat, NMES = neuromuscular electrical stimulation, PPTC = per-protocol therapy compliant

### Treatment Compliance

Table 2 summarizes the actual applied NMES therapy duration during the study for the ITT and per-protocol therapy compliant populations. In the ITT population, 50% of the patients (21/42) in the active NMES group and 33% of the patients (8/21) in the sham NMES group adhered to the full NMES therapy regimen.

**Table 2:** Actual Applied NMES Therapy Duration (Minutes) During the Extension Study

Factor	Active NMES	Sham Low-voltage NMES
ITT population		
N	40/42	21/22
Mean (SD)	2,473.7 (1,330.6)	2,111.7 (1,623.7)
Median	2,617.5	2,069.0
Range	100-5,879	4-5,721
PPTC population		
N	19/21	7/8
Mean (SD)	3,579 (702.3)	3,928 (911.9)
Median	3,380	3,657
Range	2,820-5,878	3,108 (5,694)

ITT = Intent-to-treat, NMES = neuromuscular electrical stimulation, PPTC = per-protocol therapy compliant

### Change in Visual Analog Scale Nominated Activity

**Figure 3:**
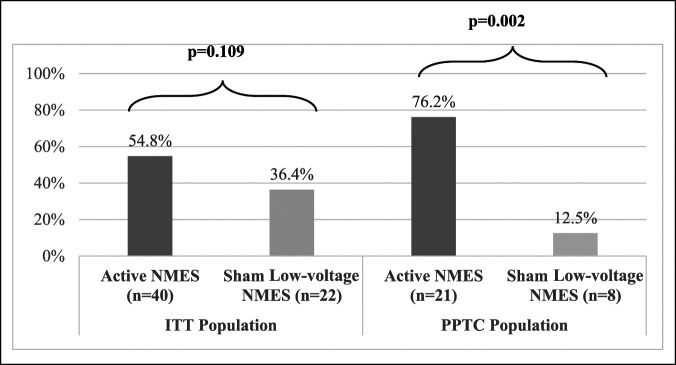
Bar graph showing the VAS Nominated Activity responder rates (≥50% improvement) for ITT and PPTC populations. ITT = intent-to-treat, NMES = neuromuscular electrical stimulation, PPTC = per-protocol therapy compliant, VAS = Visual Analog Scale

Table 3 gives the reduction in VAS Nominated Activity from baseline to the end of the parent study (week 12) and extension study (week 26 overall) for the ITT and per-protocol therapy compliant populations. The active NMES group had a larger reduction in VAS Nominated Activity than the sham NMES group for both populations. As expected, a statistically significant difference was observed between the active NMES group versus the sham NMES group (*P* = 0.020) only in the per-protocol therapy compliant population. The reduction in VAS Nominated Activity experienced by the active NMES group increased during the extension study relative to the parent study. As shown in Figure 3, a larger proportion of patients in the active NMES group experienced a minimum of 50% reduction in VAS Nominated Activity compared with the sham NMES group with the active NMES group in the per-protocol therapy compliant population having a statistically significant larger response rate than the low-voltage sham NMES group (*P* = 0.0002).

**Table 3:** Mean (SD) Percentage Change From Baseline in VAS Nominated Activity to End of the Parent Study and Extension Study

Factor	Active NMES	Sham Low-voltage NMES	*P*
Week 12 (parent study)			
ITT population	−38.5% (33.7)	−38.1% (34.9)	0.946
PPTC population	−42.8% (37.8)	−38.6% (33.5)	0.562
Week 26 (extension study)			
ITT population	−56.5% (31.6)	−43.8% (49.0)	0.555
PPTC population	−64.7% (37.7)	−28.3% (33.6)	0.020

ITT = Intent-to-treat, NMES = neuromuscular electrical stimulation, PPTC = per-protocol therapy compliant, VAS = Visual Analog Scale

### Change in Knee Pain, Stiffness, Function, and Total Knee Symptoms

**Figure 4:**
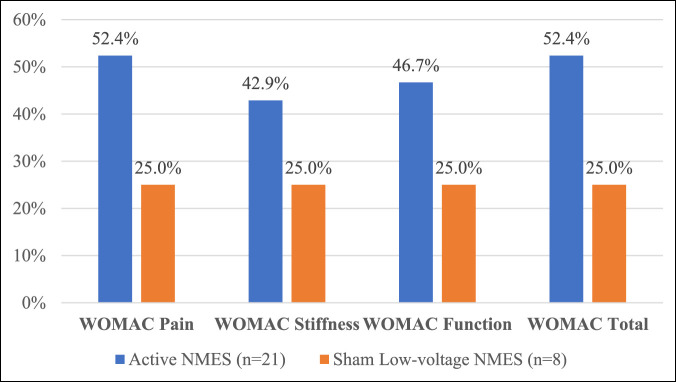
Bar graph showing the WOMAC responder rates (≥50% improvement) for PPTC population. PPTC = per-protocol therapy compliant, WOMAC = Western Ontario and McMaster Universities Arthritis Index

Larger improvements in knee pain, stiffness, and function as well as general knee health were observed in the active NMES versus sham NMES group with the largest gains made in the per-protocol therapy compliant population (Table 4). Furthermore, the per-protocol therapy compliant population achieved a larger reduction in almost all knee symptoms at the end of the extension study than was observed at the end of the parent study (Table 5). In the per-protocol therapy compliant population, approximately 50% of the patients in the active NMES group were responders on the WOMAC Pain subscale and Total scale compared with only 25% of the patients in the sham NMES group (Figure 4).

**Table 4:** Change From Baseline of Parent Study to Week 14 (Week 26 Overall) in VAS General and WOMAC Pain, Stiffness, and Function

Measure/Population	Active NMES (n = 42, ITT) (n = 21, PPTC)	Sham Low-voltage NMES (n = 22, ITT) (n = 8, PPTC)	*P*
VAS General—ITT	−52.2% (30.0)	−50.3% (35.7)	0.828
VAS General—PPTC	−56.6% (27.3)	−34.8% (36.3)	0.093
WOMAC Pain subscale—ITT	−32.5% (53.5)	−35.6% (35.7)	0.903
WOMAC Pain subscale—PPTC	−42.7% (31.9)	−21.7% (45.4)	0.148
WOMAC Stiffness subscale—ITT	−34.4% (39.7)	−37.8% (41.3)	0.840
WOMAC Stiffness subscale—PPTC	−34.4% (42.1)	−15.0% (40.0)	0.184
WOMAC Function subscale—ITT	−42.6% (38.3)	−34.1% (46.7)	0.462
WOMAC Function subscale—PPTC	−50.0% (29.5)	−14.5% (49.4)	0.026
WOMAC Total—ITT	−40.8% (37.2)	−35.3% (43.5)	0.614
WOMAC Total—PPTC	−47.4% (29.4)	−17.2% (43.5)	0.040

ITT = Intent-to-treat, NMES = neuromuscular electrical stimulation, PPTC = per-protocol therapy compliant, VAS = Visual Analog Scale, WOMAC = Western Ontario and McMaster Universities Arthritis Index

**Table 5:** Change in VAS General and WOMAC Scores at End of Parent Study (Week 12) Relative to Baseline and End of Extension Study (Week 26) Relative to for PPTC Population

Measure	Active NMES (n = 21)	Sham Low-voltage NMES (n = 8)	*P*
VAS General—Week 12	−43.4% (40.0)	−32.7% (38.5)	0.175
VAS General—Week 26	−56.6% (27.3)	−34.8% (36.3)	0.093
WOMAC Pain subscale—Week 12	−36.8% (54.7)	−26.6% (32.2)	0.038
WOMAC Pain subscale—Week 26	−42.7% (31.9)	−21.7% (45.4)	0.148
WOMAC Stiffness subscale—Week 12	−44.7% (35.6)	−17.4% (41.3)	0.002
WOMAC Stiffness subscale—Week 26	−34.4% (42.1)	−15.0% (40.0)	0.184
WOMAC Function subscale—Week 12	−40.1% (34.3)	−24.5% (34.2)	0.029
WOMAC Function subscale—Week 26	−50.0% (29.5)	−14.5% (49.4)	0.026
WOMAC Total score—Week 12	−33.7% (31.7)	−25.2% (33.7)	0.148
WOMAC Total score—Week 26	47.4% (29.4)	−17.2% (43.5)	0.040

ITT = Intent-to-treat, NMES = neuromuscular electrical stimulation, PPTC = per-protocol therapy compliant, VAS = Visual Analog Scale, WOMAC = Western Ontario and McMaster Universities Arthritis Index

### Isometric Quadriceps Strength

Isometric quadriceps strength improved in the active NMES group at week 14 compared with the baseline, although there were no statistically significant differences between the study groups. Within-groups comparison of change in isometric quadriceps strength showed that the active NMES group in the ITT population experienced a statistically significant increase in quadriceps strength at week 14 (week 26 of the parent study) compared with the parent study baseline (29%, *P* = 0.004), whereas the sham group showed no statistically significant change (23%, *P* = 0.231).

### Functional Tests

There were no statistically significant between-group differences on the TUG test or repeated chair rise test for either population from baseline of the parent study to week 14 of the extension study (Table 6).

**Table 6:** Change From Baseline of Parent Study to Week 14 (Week 26 Overall) in Timed Up and Go and Repeated Chair Rise Tests

Measure/Population	Active NMES (n = 42, ITT) (n = 21, PPTC)	Sham Low-voltage NMES (n = 22, ITT) (n = 8, PPTC)	*P*
TUG test—ITT	−15.5% (18.3)	−19.6% (22.5)	0.469
TUG test—PPTC	−11.5% (19.6)	−16.3% (18.9)	0.561
Repeated chair rise test—ITT	17.7% (38.9)	30.0% (38.5)	0.289
Repeated chair rise test—PPTC	22.6% (49.0)	23.1% (31.3)	0.753

ITT = Intent-to-treat, NMES = neuromuscular electrical stimulation, PPTC = per-protocol therapy compliant, TUG, Timed Up and Go

### Patients' Perspective

In the per-protocol therapy compliant population at week 14 (week 26 overall), 84% of the patients in the active NMES group and 50% in the sham NMES group responded either “much improved” or “very much improved” on the PGIC relative to their health status at the beginning of the study. In the per-protocol therapy compliant population, 95% of the patients in the active NMES group versus 50% in the sham group responded either “moderately satisfied” or “very satisfied” with the treatment (*P* = 0.009).

### Adverse Events

No adverse events were reported in either study group during the extension study.

## Discussion

This study demonstrates that home-based NMES therapy, when used for 26 weeks, results in clinically meaningful and statistically significant improvements in knee pain, stiffness, function, and general health compared with sham NMES treatment. As expected, the greatest magnitude of improvement was seen among patients who were fully compliant with the active NMES treatment. Although the primary end point was not achieved for the ITT population, in the per-protocol therapy compliant population, the active NMES group had a statistically significant higher response rate for VAS Nominated Activity (76.2% versus 12.5%, *P* = 0.002) and percentage reduction from baseline in VAS Nominated Activity (64.7% versus 24.3%, *P* = 0.020) compared with the sham NMES group. Consistent with these findings, the active NMES group also experienced larger and clinically meaningful improvements in self-reported knee pain, stiffness, function than the sham NMES group, with a larger therapeutic benefit observed after a total of 26 weeks versus 12 weeks of treatment.

Although isometric quadriceps strength in the active NMES group increased at week 14 compared with baseline, the gain in quadriceps strength at week 14 (week 26 overall) was lower than the gain at week 12 of the parent study (29% versus 64.7%). A possible explanation for this finding may be that five patients who achieved very high increases in quadriceps strength during the parent study were ineligible or declined to participate in the extension study. Given that quadriceps strength is 20% to 40% weaker in patients with knee OA than healthy control subjects,^6^ a 29% gain may be sufficient to normalize quadriceps strength in many patients with knee OA. Observed increases in quadriceps strength in the sham NMES group over time suggest a potential therapeutic benefit from the daily consistent application of low-voltage NMES therapy, which may have contributed to improvements in other outcomes; however, the mechanism of action and magnitude of benefits from a low-intensity NMES are unknown.

Although no standard protocol has been developed for the application of NMES to increase quadriceps strength, new guidelines recommend a treatment duration of at least 20 minutes applied 5 times per week to achieve optimal effectiveness. Because the studies which resulted in this guideline did not find that NMES, even with the recommended protocol, reduced pain by ≥30%,^28^ our protocol required more intensive treatment (two 20-minute sessions on 5 days per week) to achieve a ≥30% reduction in pain. As expected, we found that the benefits of NMES therapy were considerably greater for patients who were fully compliant with the treatment protocol. Although an adherence rate of 50% to the full protocol of the active NMES arm is somewhat disappointing, adherence to self-managed, home-based physical therapy protocols may be as low as 30%.^38^ The strongest predictors of adherence to self-managed, home-based physical therapy are intention to engage in the program, self-motivation, self-efficacy, previous adherence to exercise-based behaviors, and social support.^38^ Assessment of these domains may identify patients who are at highest risk for poor adherence to home-based NMES who may benefit from interventions that target these issues. Clinicians may also consider that some patients at particularly high risk for poor adherence to home-based NMES may fare better with supervised NMES therapy. Future research should evaluate methods to screen patients for issues associated with nonadherence and whether targeted interventions can increase adherence to home-based NMES. Because the CyMedica NMES system (CyMedica Orthopedics) captures and reports data on the actual use of treatment in real time, it should be possible to enable the system to deliver automated mobile app reminders to remind and encourage patients to complete NMES sessions, which can be tailored based on patients' patterns of NMES use or other factors.^39^

Although this study used a rigorous methodology, several limitations should be noted. First, patients in the low-voltage sham NMES group may have experienced a strong placebo response, which is well established in OA studies,^40^ and could have obscured differences between groups; however, the placebo response is strongest for assessments of pain and weaker for assessments of function.^40^ Second, some patients in the sham NMES group may have experienced some level of therapeutic benefits from the continuous use of the low-voltage NMES over 26 weeks, which is not quantifiable in conjunction with the placebo effect. Third, the size of the ITT and per-protocol therapy compliant compliant populations was relatively small, limiting power to detect between-group differences.

## Conclusions

Home-based NMES therapy is a noninvasive, conservative, and safe treatment modality that increases quadriceps strength in a painless manner, potentially providing benefits to many patients with knee OA. Patients with knee OA who used home-based NMES as prescribed over 26 weeks experienced clinically meaningful and consistent improvements in OA-related knee pain, stiffness, and function. Furthermore, the cohort of compliant patients who received the original NMES perceived the treatment as valuable with 84% reporting having experienced much or very much improvement and 95% responding that they were moderately or very satisfied with treatment. Continuous use of the NMES treatment beyond 12 weeks, for additional 14 weeks, provided additional reduction of knee pain, stiffness, and improvements in knee joint functionality at week 26.

## References

[1] Arthritis Foundation: Arthritis by the numbers/book of trusted facts & figures 2019, https://www.arthritis.org/getmedia/e1256607-fa87-4593-aa8a-8db4f291072a/2019-abtn-final-march-2019.pdf, Accessed May 5, 2021.

[2] Deshpande BR Katz JN Solomon DH : Number of persons with symptomatic knee osteoarthritis in the US: Impact of race and ethnicity, age, sex, and obesity. Arthritis Care Res (Hoboken) 2016;68:1743-1750. 27014966 10.1002/acr.22897 PMC5319385

[3] Lespasio MJ Piuzzi NS Husni ME Muschler GF Guarino A Mont MA: Knee osteoarthritis: A primer. Perm J 2017;21:16-183. 10.7812/TPP/16-183 PMC5638628 29035179

[4] Øiestad BE Juhl CB Eitzen I Thorlund JB: Knee extensor muscle weakness is a risk factor for development of knee osteoarthritis. A systematic review and meta-analysis. Osteoarthritis Cartilage 2015;23:171-177. 25450853 10.1016/j.joca.2014.10.008

[5] Slemenda C Heilman DK Brandt KD : Reduced quadriceps strength relative to body weight: A risk factor for knee osteoarthritis in women? Arthritis Rheum 1998;41:1951-1959. 9811049 10.1002/1529-0131(199811)41:11<1951::AID-ART9>3.0.CO;2-9

[6] Bennell KL Wrigley TV Hunt MA Lim BW Hinman RS: Update on the role of muscle in the genesis and management of knee osteoarthritis. Rheum Dis Clin North Am 2013;39:145-176. 23312414 10.1016/j.rdc.2012.11.003

[7] Chin C Sayre EC Guermazi A : Quadriceps weakness and risk of knee cartilage loss seen on magnetic resonance imaging in a population-based cohort with knee pain. J Rheumatol 2019;46:198-203. 30275263 10.3899/jrheum.170875

[8] Glass NA Torner JC Frey Law LA : The relationship between quadriceps muscle weakness and worsening of knee pain in the MOST cohort: A 5-year longitudinal study. Osteoarthritis Cartilage 2013;21:1154-1159. 23973125 10.1016/j.joca.2013.05.016 PMC3774035

[9] Slemenda C Brandt KD Heilman DK : Quadriceps weakness and osteoarthritis of the knee. Ann Intern Med 1997;127:97-104. 9230035 10.7326/0003-4819-127-2-199707150-00001

[10] Segal NA Torner JC Felson D : Effect of thigh strength on incident radiographic and symptomatic knee osteoarthritis in a longitudinal cohort. Arthritis Rheum 2009;61:1210-1217. 19714608 10.1002/art.24541 PMC2830551

[11] Hurley MV: The role of muscle weakness in the pathogenesis of osteoarthritis. Rheum Dis Clin North Am 1999;25:283-298; vi. 10356418 10.1016/s0889-857x(05)70068-5

[12] Kim D Park G Kuo LT Park W: The effects of pain on quadriceps strength, joint proprioception and dynamic balance among women aged 65 to 75 years with knee osteoarthritis. BMC Geriatr 2018;18:245. 30332992 10.1186/s12877-018-0932-y PMC6192068

[13] O'Reilly SC Jones A Muir KR Doherty M: Quadriceps weakness in knee osteoarthritis: The effect on pain and disability. Ann Rheum Dis 1998;57:588-594. 9893569 10.1136/ard.57.10.588 PMC1752483

[14] Ruhdorfer A Wirth W Eckstein F: Relationship between isometric thigh muscle strength and minimum clinically important differences in knee function in osteoarthritis: Data from the osteoarthritis initiative. Arthritis Care Res (Hoboken) 2015;67:509-518. 25303012 10.1002/acr.22488 PMC4376605

[15] Bannuru RR Osani MC Vaysbrot EE : OARSI guidelines for the non-surgical management of knee, hip, and polyarticular osteoarthritis. Osteoarthritis Cartilage 2019;27:1578-1589. 31278997 10.1016/j.joca.2019.06.011

[16] Kolasinski SL Neogi T Hochberg MC : 2019 American College of Rheumatology/Arthritis Foundation guideline for the management of osteoarthritis of the hand, hip, and knee. Arthritis Care Res (Hoboken) 2020;72:220-233. 10.1002/art.41142 PMC10518852 31908163

[17] Pelland L Brosseau L Wells G : Efficacy of strengthening exercises for osteoarthritis (part I): A meta-analysis. Phys Ther Rev 2004;9:77-108.

[18] Roddy E Zhang W Doherty M: Aerobic walking or strengthening exercise for osteoarthritis of the knee? A systematic review. Ann Rheum Dis 2005;64:544-548. 15769914 10.1136/ard.2004.028746 PMC1755453

[19] Kanavaki AM Rushton A Efstathiou N : Barriers and facilitators of physical activity in knee and hip osteoarthritis: A systematic review of qualitative evidence. BMJ open 2017;7:e017042. 10.1136/bmjopen-2017-017042 PMC5770915 29282257

[20] Laufer Y Shtraker H Elboim Gabyzon M: The effects of exercise and neuromuscular electrical stimulation in subjects with knee osteoarthritis: A 3-month follow-up study. Clin Interv Aging 2014;9:1153-1161. 25083133 10.2147/CIA.S64104 PMC4108455

[21] Petterson SC Barrance P Buchanan T Binder-Macleod S Snyder-Mackler L: Mechanisms underlying quadriceps weakness in knee osteoarthritis. Med Sci Sports Exerc 2008;40:422-427. 18379202 10.1249/MSS.0b013e31815ef285 PMC3573845

[22] Sheffler LR Chae J: Neuromuscular electrical stimulation in neurorehabilitation. Muscle Nerve 2007;35:562-590. 17299744 10.1002/mus.20758

[23] Bistolfi A Zanovello J Ferracini R : Evaluation of the effectiveness of neuromuscular electrical stimulation after total knee arthroplasty: A meta-analysis. Am J Phys Med Rehabil 2018;97:123-130. 29016401 10.1097/PHM.0000000000000847

[24] Hauger AV Reiman MP Bjordal JM Sheets C Ledbetter L Goode AP: Neuromuscular electrical stimulation is effective in strengthening the quadriceps muscle after anterior cruciate ligament surgery. Knee Surg Sports Traumatol Arthrosc 2018;26:399-410. 28819679 10.1007/s00167-017-4669-5

[25] Delanois R Sodhi N Acuna A Doll K Mont MA Bhave A: Use of home neuromuscular electrical stimulation in the first 6 weeks improves function and reduces pain after primary total knee arthroplasty: A matched comparison. Ann Transl Med 2019;7(suppl 7):S254. 31728378 10.21037/atm.2019.09.150 PMC6829003

[26] Klika AK Yakubek G Piuzzi N Calabrese G Barsoum WK Higuera CA: Neuromuscular electrical stimulation use after total knee arthroplasty improves early return to function: A randomized trial. J Knee Surg 2022;35:104-111. 32610358 10.1055/s-0040-1713420

[27] Labanca L Rocchi JE Laudani L : Neuromuscular electrical stimulation superimposed on movement early after ACL surgery. Med Sci Sports Exerc 2018;50:407-416. 29059108 10.1249/MSS.0000000000001462

[28] Novak S Guerron G Zou Z Cheung G Berteau JP: New guidelines for electrical stimulation parameters in adult patients with knee osteoarthritis based on a systematic review of the current literature. Am J Phys Med Rehabil 2020;99:682-688. 32167955 10.1097/PHM.0000000000001409

[29] Nussbaum EL Houghton P Anthony J Rennie S Shay BL Hoens AM: Neuromuscular electrical stimulation for treatment of muscle impairment: Critical review and recommendations for clinical practice. Physiother Can 2017;69:1-76. 10.3138/ptc.2015-88 PMC5683854 29162949

[30] de Oliveira Melo M Aragão FA Vaz MA: Neuromuscular electrical stimulation for muscle strengthening in elderly with knee osteoarthritis: A systematic review. Complement Ther Clin Pract 2013;19:27-31. 23337561 10.1016/j.ctcp.2012.09.002

[31] Walls RJ McHugh G O'Gorman DJ Moyna NM O'Byrne JM: Effects of preoperative neuromuscular electrical stimulation on quadriceps strength and functional recovery in total knee arthroplasty. A pilot study. BMC Musculoskelet Disord 2010;11:119. 20540807 10.1186/1471-2474-11-119 PMC2896350

[32] Pal S Chughtai M Sultan AA : Impact of neuromuscular electrical stimulation (NMES) on 90-day episode costs and post-acute care utilization in total knee replacement patients with disuse atrophy. Surg Technol Int 2017;31:384-388. 29316600

[33] Bruce-Brand RA Walls RJ Ong JC Emerson BS O'Byrne JM Moyna NM: Effects of home-based resistance training and neuromuscular electrical stimulation in knee osteoarthritis: A randomized controlled trial. BMC Musculoskelet Disord 2012;13:118. 22759883 10.1186/1471-2474-13-118 PMC3493368

[34] Gaines JM Metter EJ Talbot LA: The effect of neuromuscular electrical stimulation on arthritis knee pain in older adults with osteoarthritis of the knee. Appl Nurs Res 2004;17:201-206. 15343554 10.1016/j.apnr.2004.06.004

[35] Sax OC Gesheff MG Mahajan A : A novel mobile app-based neuromuscular electrical stimulation therapy for improvement of knee pain, stiffness, and function in knee osteoarthritis: A randomized trial. Arthroplasty Today 2022;15:125-131. 35514364 10.1016/j.artd.2022.03.007 PMC9062361

[36] Bellamy N Buchanan WW Goldsmith CH Campbell J Stitt LW: Validation study of WOMAC: A health status instrument for measuring clinically important patient relevant outcomes to antirheumatic drug therapy in patients with osteoarthritis of the hip or knee. J Rheumatol 1988;15:1833-1840. 3068365

[37] Dworkin RH Turk DC Wyrwich KW : Interpreting the clinical importance of treatment outcomes in chronic pain clinical trials: IMMPACT recommendations. J Pain 2008;9:105-121. 18055266 10.1016/j.jpain.2007.09.005

[38] Essery R Geraghty AW Kirby S Yardley L: Predictors of adherence to home-based physical therapies: A systematic review. Disabil Rehabil 2017;39:519-534. 27097761 10.3109/09638288.2016.1153160

[39] Stevens T McGinnis RS Hewgill B : A cyber-physical system for near real-time monitoring of at-home orthopedic rehabilitation and mobile-based provider-patient communications to improve adherence: Development and formative evaluation. JMIR Hum Factors 2020;7:e16605. 32384052 10.2196/16605 PMC7248795

[40] Huang Z Chen J Hu QS : Meta-analysis of pain and function placebo responses in pharmacological osteoarthritis trials. Arthritis Res Ther 2019;21:173. 31307506 10.1186/s13075-019-1951-6 PMC6631867

